# Chronicles of Involuntary Career Changes: A Qualitative Longitudinal Analysis

**DOI:** 10.1177/10690727241289126

**Published:** 2024-10-01

**Authors:** Caroline Éliane Brazier, Jonas Masdonati, Michaël Parmentier

**Affiliations:** 1 Research Center in Vocational Psychology and Career Counseling (CEPCO), Institute of Psychology, 27213 University of Lausanne, Lausanne, Switzerland; 2 Swiss National Centre of Competence in Research LIVES – Overcoming Vulnerability: Life Course perspectives (NCCR LIVES), 27213 University of Lausanne, Lausanne, Switzerland; 3 HEC Liège—Management School, 82481 University of Liège, Liège, Belgium

**Keywords:** involuntary career change, career transition, subjective career, qualitative longitudinal research, synchronicity

## Abstract

Although extensive research on career transitions exists, little is known about the challenges and processes of involuntary career change. Building on Savickas’s theoretical framework of objective and subjective careers, we investigated the subjective experiences of involuntary career changes while apprehending their objective unfolding. Following a longitudinal qualitative design, we conducted two waves of semistructured interviews with 18 participants who had been forced to change careers due to health issues, migration, or saturated labor market in Switzerland. A four-step temporal thematic analysis covering case description, case comparison, case processes analysis, and processes comparison highlighted a spectrum of career change processes. At one end, individuals experienced synchronous progressions and regained meaning and control over their career combined with a rather linear status sequence. Conversely, some individuals underwent asynchronous developments consisting of either modest objective steps that instigated meaningful subjective experiences or status stagnation, leading to a loss of control and meaning. These findings underscore the variety of involuntary career change processes and unveil synchronicity as a key temporal element in involuntary career change processes. Implications for research and practice are discussed.

## Introduction

Contemporary career paths are characterized by frequent and diversified career transitions (De Vos et al., 2021; Sullivan & Al Ariss, 2021). Alongside socially expected transitions, such as moving from school to work (e.g., Schoon & Heckhausen, 2019) and from work to retirement (e.g., Froidevaux et al., 2018), other career transitions (e.g., unemployment, lateral or downward mobility, sick leaves; Bidart, 2019; Lipshits-Braziler & Gati, 2019) are situation-dependent and less predictable. In some cases, unexpected events or circumstances lead to a mismatch between individuals’ skills and occupations, forcing them to abandon their current career and find a new occupation, that is, to go through an involuntary career change (Masdonati et al., 2022).

Also referred to as an occupational change, a *career change* implies a shift to a new occupation that does not align with the previous occupation, thus requiring new formal or informal learning (Carless & Arnup, 2011). This change can be triggered intentionally by workers or forced by reasons beyond their control (Fouad & Bynner, 2008). Because of the significant hassles it poses for individuals, such as need for retraining, human-capital investment, and a possible loss of income, a career change, especially an involuntary one, has been described as one of the most demanding and complex transitions people might go through in their working life (Carless & Arnup, 2011; Medici et al., 2020). Indeed, involuntary career changers face several obstacles, such as a lack of time, information, and support (Fouad & Bynner, 2008). They sometimes encounter institutional barriers, such as limited access to training and career support services or rigidities and misunderstandings from employers (Masdonati et al., 2022). In addition, involuntary career changes can jeopardize people’s career trajectories. According to Bachmann et al. (2020), individuals who change occupations are likely to have a lower income and experience downward mobility, these negative effects being stronger for workers changing involuntarily. Therefore, an involuntary career change can be considered a career shock (Akkermans et al., 2018) that disrupts careers and affects individual trajectories lastingly.

Involuntary career changes inherently involve a temporal dimension. First, the process of moving from an old to a new occupation can be time consuming (Hennekam & Bennett, 2016). Between quitting a former career and entering a new one, individuals may go through various statuses (e.g., unemployed, student, trainee, part-time employee), implying microtransitions and efforts to implement their career plan. In contrast with voluntary career change (e.g., Barclay et al., 2011), however, involuntary career change is unintentional and less predictable, and might therefore not follow a unified, stepwise progression. Second, adjusting to a new career is a prolonged journey marked by continuous learning, identity work, and meaning making through time (Olry-Louis et al., 2022). This encompasses decision-making challenges, sacrifices, the reevaluation of personal and professional goals, and the development of new professional identities (Ahn et al., 2017; Masdonati et al., 2022; Motulsky, 2010; Murtagh et al., 2011). Indeed, as individuals navigate these changes, they engage in continuous meaning making, attempting to weave a coherent narrative that bridges their past self with potential future selves (Kulkarni, 2020). This narrative construction is not static but evolves over time, reflecting the ongoing identity work and the subjective valuation of one’s career trajectory (Wise & Millward, 2005). The temporal aspect is therefore crucial in understanding the full spectrum of challenges and experiences associated with involuntary career change.

Although extensive research has been conducted on socially expected transitions, less is known about how individuals experience less predictable career transitions (Sullivan & Al Ariss, 2021), and among those, involuntary career change remains understudied. The sporadic studies on this topic suggest that three main factors seem to trigger an involuntary career change: a health problem forcing to stop working in a given sector (e.g., Baldridge & Kulkarni, 2017); a job loss in a saturated sector, hindering reentry into the job market in that same sector (e.g., Gardiner et al., 2009); and migration to countries that do not recognize qualifications and experiences obtained in the country of origin (e.g., Palic et al., 2023). In addition, existing research is fragmented and mainly focused on particular occupational groups, such as artists (e.g., Hennekam & Bennett, 2016), veterans (e.g., Haynie & Shepherd, 2011; Kulkarni, 2020), and athletes (e.g., Arvinen-Barrow et al., 2018). Consequently, little is known about involuntary career change experiences, regardless of the specific occupations with which they are associated. Finally, the literature that addresses involuntary job exits due to job loss (e.g., Lent et al., 2023) does not further explore how people transition to a new career. Thus, we also lack a comprehensive understanding of involuntary career change processes. Considering the criticality of involuntary career change in career paths (Bachmann et al., 2020), the time often needed to go through them (Hennekam & Bennett, 2016), and the array of obstacles potentially encountered (Fouad & Bynner, 2008; Masdonati et al., 2022), this lack of knowledge is problematic. Indeed, it hinders implementation of relevant policies and appropriate career support to help individuals attain a satisfactory career.

### Career Construction Theory and Career Change

We build on Savickas’s (2000) career construction theory as a suitable theoretical framework for comprehensively capturing the complex and temporal nature of involuntary career change. This framework considers careers not merely as sequences of events but as dynamic, individualized journeys influenced by personal and environmental factors (Savickas, 2002, 2020). According to Savickas (2020), workers construct a subjective career by identifying a plot from their objective career. The objective career (OC; Savickas, 2002) refers to the sequences of occupations and occupational statuses throughout an individual’s life course. The subjective career (SC) refers to the process through which people construct significations and make sense of their experience. This process can lead people to deliberate about their vocational identity (Savickas, 2020) and engage in identity work with the aim of repairing, reforming, maintaining, or revising their identity (Ibarra & Barbulescu, 2010). Savickas’ (2020) recent work also emphasized the key role of the environment in the intertwining and dynamic evolution of objective and subjective careers, leading him to maintain that “individuals build a self from the outside in, not the outside out” (p. 166). This observation argues in favor of exploring how external contexts are subjectively integrated.

According to Savickas (2002), the career construction theory is suited to study transitions occurring “each time an individual’s career is destabilized by socioeconomic and personal events such as illness and injury, plant closings and company layoffs, and job redesign and automation” (p. 156 ), which can result in involuntary career change. In this sense, involuntary career changes can be conceived as markers of a forced modification of a worker’s occupational status (OC). They imply leaving an initial occupation, going through intermediate situations—possibly benefiting from support structures—and integrating a new occupation. This status change can be more or less prolonged and take several forms (Mulhall, 2014; Wise & Millward, 2005). For example, some individuals undergo internships, others return to school or vocational training, while others quickly secure new positions or become entrepreneurs. From the SC perspective, an involuntary career change might be experienced as an opportunity to take control of one’s life or, on the contrary, as a source of job insecurity (Bachmann et al., 2020). The subjective meaning of an involuntary career change may also depend on its perceived social desirability, radicality, skills transferability, and reversibility (Masdonati et al., 2017; Zacher, 2019). In sum, objective and subjective perspectives need to be articulated to fully understand involuntary career change experiences and processes (Olry-Louis et al., 2022).

### Current Study

Although considerable research has been conducted on socially expected career transitions (Sullivan & Al Ariss, 2021), involuntary career change and its temporal and processual complexity have been understudied. This oversight is notable given the impact such transitions can have on individuals’ future career trajectories and well-being (Bachmann et al., 2020; Carless & Arnup, 2011; Medici et al., 2020). The general aim of this study was to provide an understanding of involuntary career change experiences and processes. More specifically, drawing on career construction theory (Savickas, 2000) and considering the temporal dimension of career transitions (Olry-Louis et al., 2022), we investigated how the process of involuntary career change unfolds over time from a subjective and an objective perspective. In order to address both subjective and temporal processes, we implemented a qualitative and longitudinal design. Such a design is suited to investigating “change in the making” (Neale & Tarrant, 2024, p. 53) and to understand career change processes as individualized experiences. Consequently, we conducted 2-wave qualitative longitudinal research (QLR; Neale, 2021; Saldaña, 2003) based on semistructured interviews with people forced to change career due to (1) health issues, (2) unemployment in saturated sectors, and (3) non-recognition of qualifications following migration.

The study took place in Switzerland, where career mobility is relatively high compared to other European countries (Bachmann et al., 2020). In 2018, one in five workers had left their jobs, with half of them experiencing a change of occupation and one fifth of them reporting a potential involuntary reason to leave their employment, such as dismissal, accident, or end of contract (Federal Statistical Office, 2020). However, these figures are only approximate regarding the intentionality of career change. Moreover, in Switzerland as elsewhere, no studies have been conducted on the experiences and processes involuntary career change.

## Method

### Research Design

QLR is a research strategy that provides access to subjective changes and processes through time, especially during major life events (Neale & Tarrant, 2024; Saldaña, 2003; Vogl et al., 2018). It is therefore appropriate to capture the evolving nature of career changes, providing insights into their temporal development and the adjustment processes that occur with them. Despite its popularity in social and health sciences (e.g., Audulv et al., 2022; Treanor et al., 2021), QLR remains uncommon in vocational psychology (Farr & Nizza, 2019; McCoy, 2017; Vogl et al., 2018).

Based on Ponterotto’s (2005) classification and in line with our theoretical framework (Savickas, 2002), our study is rooted in a constructivist paradigm. This paradigm emphasizes the importance of individual interpretation and meaning-making in understanding realities (Ponterotto, 2005), recognizing their socially constructed and subjective nature. Indeed, since we are interested in understanding how people subjectively signify their objective careers, our research focuses on shared constructions of meaning.

### Procedure

To recruit participants in their early process of an involuntary career change, we contacted 13 public and semipublic institutions active in adult vocational rehabilitation and occupational integration in Switzerland. The institutions provided support for the three most common profiles of involuntary career changers identified in a previous study conducted in the same context (Masdonati et al., 2022), that is, workers having to change career because of health, labor market saturation, and migration issues. Upon institutional managers’ approval, our research team worked with career counselors and job coaches to introduce the study to potential participants. Upon participants’ voluntary informed consent, online interviews were conducted (T1). At the end of the interview, participants were asked for permission to be contacted again 1 year later for a follow-up interview (T2).

Given the emotional aspects of involuntary career changes, we paid particular attention to participants’ well-being during interviews, inquiring about their emotions and encouraging them to share their thoughts and concerns. Following Thomson and Holland (2003), we also explored participants’ reflexivity during their participation in our research. To ensure ethical collaboration and relational continuity between T1 and T2, participants were sent an intermediary report (Frésard et al., 2022) . The interviews lasted between 66 and 146 minutes (*M* = 99) at T1 and between 44 and 161 minutes (*M* = 94) at T2. Four researchers (i.e., the three authors and a PhD student) conducted the interviews, and the three authors performed the analyses. The first author experienced a voluntary career change, moving from teaching to psychology; the second author experienced geographical mobility without changing career; and the third author did not go through any career change. The authors’ university ethics committee approved the study (N°C_SSP_052021_00003).

### Participants

Participants were 18 career changers from the French-speaking part of Switzerland, nine women and nine men, aged from 28 to 49 (*M* = 37.5, *SD* = 7.31). Inclusion criteria were (a) having begun an involuntary career change within the past year, (b) intermediate French, and (c) participation in both waves of interviews. Participants were recruited from a larger research project, in which the research team met 48 career changers in a first wave of interviews and 33 in a second wave of interviews. The larger project encompassed cross-sectional explorations of involuntary career changers’ relational context (Masdonati et al., 2022), and career-decision-making strategies (Brazier et al., 2024). The present paper offers the first longitudinal data analysis. For the present study, we selected the first career changers who took part in the two waves of interviews in the larger project, applying quotas for gender and the trigger of career change. This resulted in a sample of 18 participants, nine women, and nine men, changing careers because of health issues (*n* = 6), a saturated labor market (*n* = 6), and non-recognition of qualifications after migration (*n* = 6), and interviewed twice over a one-year period. Data saturation in relation to the research objectives was consensually agreed on among the authors per the guidelines Sim et al. (2018) established.

Table 1 shows that the participants came from various occupational sectors (e.g., health, tourism, services, construction), with educational backgrounds ranging from vocational education and training to university, and held various positions, ranging from operational to managerial roles. Their geographical origins covered Switzerland (*n* = 8) as well as European (*n* = 4) and non-European countries (*n* = 6). At T1, three participants were employed or interns and 15 were seeking or receiving public support, including social welfare for unemployed people (*n* = 5), support for people with invalidity (*n* = 6), social welfare following unemployment support (*n* = 1) and social welfare for migrants (*n* = 6). Participants also received career counseling and assistance with employment, retraining, or internships. At T2, 10 participants’ status changed, including a return to work (part-time *n* = 2 and full-time employment *n* = 5), retraining (*n* = 2), and internships (*n* = 1).

**Table 1.** Demographic characteristics of participants.

Name	Age	Gender	Origin	Career change trigger	Previous occupation	Statuses T1	New career plan in T1	Statuses T2
Emilie	29	F	Swiss/Spanish	LMS	Commercial manager	SWU	Coaching entrepreneur	SWU
William	41	M	Swiss/British	LMS	Flight coordinator	SWU	Not determined	PTE, customer services
Nancy	41	F	British	LMS	Librarian	U	Education manager	U
Marie	42	F	Swiss	LMS	Bookseller	SWU	Librarian	PTE bookseller, RET librarian
Sarah	45	F	Swiss	LMS	Executive assistant	FTE	HR manager	FTE, HR manager
Valentina	47	F	Italian	LMS	Art teaching assistant	SW	Sustainable fashion entrepreneur	PTE, fashion upcycler
Kevin	29	M	French	H	Hairdresser	U, SPI	HR assistant	FTE, HR assistant
Frédéric	29	M	Swiss	H	Carpenter	SWU	Geomatician	SPI, RET geomatician
Jean	31	M	Swiss	H	Money courier	SPI, INT	Security manager	FTE, security manager
Josefa	35	F	Portuguese	H	Head housekeeper	U	Administrative assistant	U
Véronique	44	F	Swiss	H	Hairdresser	SPI, INT	Administrative assistant	SPI, INT, HR assistant
Anna	49	F	Swiss	H	Nurse	U, SPI	Care coordinator	U, SPI
Idriss	28	M	Syrian	M	Pilot	SWM	Manager in business	SWM
Said	32	M	Somalian	M	Printer	SWM, PTE	Cleaning agent	FTE, cleaning agent
Erkan	32	M	Turkish	M	Police commissioner	SWM, INT	Social worker	SWM, RET social worker
Olga	32	F	Belarussian	M	Nurse	SWM	Dental assistant	FTE, translator
Selim	44	M	Syrian	M	Dentist surgeon	SWM, PTE	Taxi driver	SWM, PTE, taxi coordinator
Tarek	45	M	Sudanese	M	International reporter	SWM, INT	PTE sales, PTE reporter	SWM, INT media

*Notes.* Pseudonyms are used for the participants’ names. F = female, M = male, LMS = labor market saturation, H = health, M = migration. Statuses: FTE = full-time employed, PTE = part-time employed, INT = internship, RET = retraining, U = Unemployed, SW = social welfare following unemployment, SPI = support for people with invalidity, SWU = social welfare for unemployed, SWM = social welfare for migrants.

### Interview Protocols

The guidelines for semi-structured interviews were consensually designed within a larger study on involuntary career change (see Appendix A). At T1 and T2, the interview guidelines comprised seven sections: (a) sociodemographic information; (b) career path; (c) career change process; (d) personal, social, and professional identities; (e) resources and barriers; (f) relationship to work; and (g) relationship to training. For the present study’s purposes, we mainly focused on the first three sections, which were tailored to describe participants’ careers and career change processes. The first section concerned demographic information, education level (T1), and any changes over the last year (T2). The second section concerned participants’ career history, key extraprofessional events, last employment, current situation (T1), and the evolution of the career change experience and situation over the last year (T2). In both interview phases, this part concluded with questions on their future career trajectory, ideals, and future selves. The third section focused on the reasons, perceived control, intentionality, continuity, importance, and reversibility of the career change, including participants’ expectations of, reactions to, and emotions regarding it. Questions on the perceived evolution of these aspects were added at T2. Although similar to the T1 guide, the T2 guide considered recommendations for qualitative longitudinal designs (Hermanowicz, 2013). First, we adopted two ways of exploring changes: either by asking the same questions at T1 and T2 or by addressing participants’ perceived changes. Second, alongside objective status sequences, we explored subjective temporalities concerning the perceived beginning and end of career change, personal rhythms, and tempos.

### Data Analysis

To examine how the process of involuntary career change unfolds over time from a subjective and an objective perspective, we implemented a *temporal thematic analysis*, a specific QLR strategy consisting of integrating cases, themes, and processes (Neale, 2021; Vogl et al., 2018). Following the recommendations of Neale (2021) and Vogl et al. (2018), we designed a 4-step analysis procedure, combining within-case and cross-case comparisons: (1) case description, (2) case comparison, (3) case process analysis, and (4) processes comparison. Divergences among the research team were resolved by returning to the raw data to find consensus.

#### Case Description

In the first step, we wrote pen portraits for each participant based on the T1 and T2 interviews to provide a detailed description of each case, organized chronologically (Neale, 2021, p. 288; Sheard & Marsh, 2019). This technique consisted of editing a close and thick description of each interview (five to six pages), using Sheard and Marsh’s steps (2019): (1) understanding and defining what to focus on (i.e., the first three sections of the interviews); (2) designing a basic structure relevant to the dataset (i.e., the questions asked in these sections); (3) populating the content (i.e., summarizing participants’ answers to each question); (4) interpreting the data (i.e., drafting provisional and large themes). We closely adhered to participants’ narratives, incorporating quotes to highlight specific experiences or ambivalences (Thomson & Holland, 2003). The research team coedited the first three pen portraits, and the first author edited the remaining portraits.

#### Case Comparison

In the second step, we used a grid analysis technique to compare each case across time, integrating thematic and temporal themes (Neale, 2021). Based on the pen portraits and primary data, we collectively identified themes for each case at T1 and T2. The research team independently reviewed all portraits and met to generate themes. After thorough discussions of all pen portraits, each researcher explored the objective (OC) and subjective career (SC) of three cases, identified themes, and compared them with the other researchers. Once a first set of themes was agreed upon, the researchers individually analyzed three additional cases and compared their analyses. Upon reaching a consensus on the relevant themes, the researchers independently analyzed an additional case to ensure consistent coding. Through this process, the research team reached an agreement on a set of themes that encompassed all participants’ OC and SC experiences in “a broad brush” (Neale, 2021, p. 297). Six themes were identified to characterize OCs: (a) previous career path, (b) triggers of the involuntary career change, (c) statuses, (d) career support, (e) career plans, and (f) critical extraprofessional events. Seven themes were identified to characterize SCs: (a) subjective previous career path, (b) career anticipations, (c) vocational identification, (d) perceived career control, (e) subjective experience of career change, (f) social roles, and (g) subjective temporality. Table 2 provides definitions and exemplary quotes for each theme. The researchers met again to ensure mutual understanding and theme refinement. They retested the themes on the first three cases and produced participant’ grids with themes in rows and times in columns (Neale, 2021). The first author then applied the grid to the 11 remaining cases and submitted her analysis to the team.

**Table 2.** Objective and subjective career themes, definitions, and examples.

	Themes	Definition	Examples
Objective career	(OC1) previous career path	Career path key steps, such as training, employment sequences, past career transitions, and changes.	I’ve been working since Jan. I’m doing a replacement in a bookstore at 40%…. Now I’ve started an internship … at [Swiss university]. (Marie, T2)
	(OC2) triggers of the involuntary career change	Main reason for career change, to which “secondary” reasons may be added.	The career change was imposed … by this allergy. (Kevin, T2)
	(OC3) statuses	The current status (es) endorsed by the person.	[I am] still at [SPI integration measure] … and now I’m in an internship … for three days a week, and I have one day … at the association. (Véronique, T2)
	(OC4) career support	Institutional help the person benefits from.	[With the professional insertion services mandated by the SPI], it began the work of studies of my case … my capacities … my centers of interest (Jean, T1)
	(OC5) career project	Concrete professional plan.	I started to think about what I can do in Switzerland, how I can retrain…. And then, my job counselor suggested … the social field. There is a social work auditor program at the university. (Erkan, T1)
	(OC6) critical extraprofessional events	Key personal events that affected career change.	[Obtaining my residence permit], my passport … changes a lot … [so I can get my pilot’s license]. (Idriss, T1)
Subjective career	(SC1) subjective previous career path	The participant’s subjective perspective on their career path prior to career change.	[My career path is the image of a fall]. I started … at the top. I got the [nursing studies] diploma. Then I come to Switzerland…. It fell, all that…. Now I hope I start this apprenticeship…. It will go up a bit more. (Olga, T1)
	(SC2) career anticipations	Future-oriented career thoughts and feelings, including potential future career, training, or life plans.	I’m not autonomous. I’m with [SWM]. I don’t like it because I don’t feel free…. The goal … it’s not always to stay in cleaning…. I’d like to do another training like … bus driver. I’d like to … progress. (Said, T1)
	(SC3) vocational identification	The ways the participant identifies themselves with their occupation or career, interprets how the career change influences such identification, and constructs a new vocational identity.	Forgetting about the air tourism business, but it wasn’t easy. It’s not easy.… So I did [various] missions.… [Now], it’s true that I’m questioning … will I go back [to aviation]?… This passion, I have a little difficulty in completely forgetting it. (William, T2)
	(SC4) perceived career control	Sense of control, choice, and agency in the process of career change.	On the one hand … [the career change] was imposed because the layoff was not my choice. But on the other hand, I have the impression that I have the choice in what type of career change I will go into. (Emilie, T1)
	(SC5) subjective experience	The lived experience of career change, including the associated emotions and meanings.	I had the anger . Why can’t I do it?… I let go…. I’m not going to be a nurse like I was before. Maybe it’s going to be better than I think. (Anna, T1)
	(SC6) social roles	The ways the participant articulates and integrates their social roles throughout the career change.	I started to look for a job, but … my son was very young, and in Switzerland, it’s difficult to find the childcare and all that. (Nancy, T1)
	(SC7) subjective temporality	Any element of temporality that colors the career change experience, such as rhythms, cycles, or timing.	This year, it’s 3rd time I applied [for entry into dentistry college to do the equivalencies]…. … Wasted wasted time, because every year it passes, … I forget something in my professional [skills]. (Selim, T2)

*Notes*. Pseudonyms are used for participants’ names. SPI = support for people with invalidity, SWM = social welfare for migrant.

#### Case Processes Analysis

The third step involved analyzing the T1–T2 change processes following a process tracking technique (Neale, 2021; Saldaña, 2003), based on grids and primary data. This consisted of exploring and connecting each case’s T1 and T2 grids to uncover patterns of processes and changes, called *processual threads*. Processual questions were applied to the grids, such as, “What emerges, increases, decreases, ceases through time?” “What are the sequences, phases, rhythms, patterns, or tempos?” “What are drivers and deterrents of change?” and “What are the continuities or ruptures?” (see Neale, 2021, pp. 326–327). Each researcher first analyzed an initial common case, discussed it with the team, and then individually analyzed a second case. The team analyzed four more cases together; the remaining eight were individually analyzed by team members. This led to the identification of a unique processual thread for each participant.

#### Processes Comparison

In the fourth step, we compared the processual threads across cases to identify common configurations of unfolding of OCs and SCs (Neale, 2021). The researchers independently grouped cases with similar processual threads and then collectively agreed on clusters. Following Neale (2021), we sought to develop a typology of processes, labeling and defining clusters that capture the articulation of OC and SC through time, ranging from complete harmony to full discordance. These articulations reflect how individuals interpret (SC) their concrete career unfolding (OC). Finally, the first author identified one emblematic case for each type and presented the rationale for her choice to the research team for validation. A case was deemed emblematic when its processual threads closely aligned with the cluster it belonged to. We also ensured diversity among emblematic cases in gender and reasons for career change.

## Results

We identified two types of involuntary career change unfolding; the first is divided into two subtypes, and the second into three subtypes (see Table 3). Below, we provide a detailed description of each type and its subtypes, including their main characteristics, and how OCs and SCs specifically evolve through time. We also present emblematic cases that best illustrate each subtype.

**Table 3.** Types and Subtypes of articulated deployments of subjective and objective careers triggered by an involuntary career change.

Types of process	Subtypes of process	Objective career	Subjective career	Participants
(1) Synchronous progressions	(1a) Harmonious deployment	Movement toward employment	Fulfillment, control, and continuity, optimistic and enthusiastic projections	Jean, Kevin Marie, Sarah, Erkan
	(1b) Laborious improvement	Improvement trend with challenges	Moderate satisfaction, perceived difficulties due to trials and errors	Véronique, Saïd, Olga, Frédéric
(2) Asynchronous developments	(2a) Meaningful recovery	Slight progress through unstable positions	Meaningful progress, increased positivity, hope, and sense of agency Career change in elaboration	Nancy, Valentina, Tarek
	(2b) Meaningful explorations	Stagnation with multiple microtransitions	Active meaning making Gaining experience and self-knowledge Career change in elaboration	William, Emilie
	(2c) Vocational tenacity	Status quo and failed attempts to reintegrate employment	Dissatisfaction Ideations of return to previous career Temporary vocational compromises	Selim, Idriss, Anna, Josefa

*Notes*. Pseudonyms are used for participants’ names.

### Synchronous Progressions

For nine participants, the OC and the SC followed a synchronous progression, marked by the objective improvement of their situation (i.e., reintegrating a position in the new career) and a paralleled subjective sense of positive unfolding. This type of process comprises two subtypes. Although both subtypes implied an improvement of the career situation, their OC and SC process were distinct, one characterized by a (1a) harmonious deployment, the other by a (1b) laborious improvement.

#### Harmonious Deployment

In this subtype, five participants’ situations unfolded toward secured employment in permanent positions (OC) and were positively experienced (SC). Jean, Kevin, Marie, Sarah, and Erkan were all in the process of realizing their new career plan at diverse stages of achievement. The OC improvement was associated with a subjective feeling of fulfillment, suggesting an alignment and positive synchronization of their OC and SC. Additionally, the feeling of subjective control and continuity was reinforced. They all had enrolled in and (almost) completed a retraining program with promising career perspectives. They developed optimistic future anticipations and plans, such as prospects of further education that would consolidate their new career. They also reported that they would not go back to their previous occupation if given the chance, which illustrates their feeling of fulfillment.

Kevin (29), a former hairdresser who became an HR assistant due to an allergy, is an emblematic case of harmonious deployment. His objective situation improved: Finishing an internship in HR at T1, he was about to secure a contract for a permanent full-time position as an HR assistant and considering higher education to consolidate his new career at T2. Subjectively, Kevin experienced an increasingly positive feeling: “I take stock of the positive and negative aspects of this career change.… I really discovered that in human resources, now I was going to have much more positive [features] than I had before” (T1). When we met him a year later, this feeling had become stronger: “I’m a big winner … [the career change] still went pretty damn well…. I think [it was] an opportunity for me” (T2). Like the other participants in this subtype, Kevin narrated his experience a posteriori with increased control. At T1, he stated,

I would say [the career change] was forced because I didn’t have a choice. I think if I didn’t have this allergy, I would have my own salon. But now, once the decision was made … I would say that my career change was chosen. (T1)

A year later, Kevin evaluated his control the same way, but added, “It’s going very well.… I took back control because now I’m actually the master of my own boat” (T2). Although he encountered obstacles throughout the career change process (e.g., a swift return to unemployment), Kevin was proud of his path and its outcomes. Already at T1, he reported that he would probably not go back to his previous career, even if he could: “Even if tomorrow we say, ‘Well, we have a miracle pill, and you won’t be allergic anymore. You can go back to hairdressing,’ I’m not sure I’ll go back, actually” (T1). A year later, he was even more convinced, and his mind was clearer: “I’m fine where I am…. No way back” (T2).

#### Laborious Improvement

In this subtype, the OC of four participants (i.e., Frédéric, Olga, Saïd, and Véronique) tended to improve and led, for example, to a return to work, entry into training, or job securitization. However, the career change’s outcome was not completely stabilized and was tainted by more challenges and difficulties than in the previous subtype. As a result, the OC tended to improve (i.e., a better employment status at T2 than T1) although not leading to permanent and secure position (i.e., a one-year fixed-term or part-time contract). Subjectively, the evolution of career change was experienced in halftone, with measured enthusiasm, and the satisfaction with the career change was nuanced.

Olga (32) is an emblematic case in the laborious improvement subtype. At T1, she was a former nurse forced to change career due to migration and the impossibility of having her diplomas recognized:

I sent to several hospitals ... many, many, many CVs.... I received answers: “No, your diploma doesn’t work in Switzerland.” What can I do with my diploma? Nothing, just throw it away, trash can.... I didn’t want to ask for social aid and all that.… I can’t find a job. (T1)

At T1, Olga had found a vocational training position as a dental assistant after several unsuccessful applications. When we met with her at T2, she had to quit her training due to moral harassment and irregularities. Because she could not find a new position, she volunteered and ended up with a new job as a translator and receptionist: “[The migrant reception institutions] were always looking for a volunteer who could translate from [her mother language] to French…. They offered me a job, a position … so I started to work as a receptionist and dispatcher assistant” (T2). Although her situation was becoming more stable, her job only lasted 1 year and could be subject to an abrupt termination. Although Olga’s new position represented an improvement of her OC and SC, she would have preferred to be considered as a social worker: “[This job,] it’s wonderful for me…. I do a lot of things as a social worker, but when I applied to be a social worker, I was turned down every time” (T2). For these reasons, she considered her career change with tempered enthusiasm.

### Asynchronous Developments

For nine participants, OC and SC followed an asynchronous development, meaning that the evolution of OC and SC after career change were not congruent. This type comprised three subtypes: (2a) meaningful recovery, (2b) meaningful explorations, and (2c) vocational tenacity.

#### Meaningful Recovery

In this subtype, slight signs of OC progression were present. Although rare, provisional, or unsteady, these signs were perceived as meaningful at the subjective level. Three participants (i.e., Nancy, Tarek, and Valentina) were engaged in one or two provisional career experiences that maintained hope and agency. Their OC unfolding was characterized by a change of employment status through one or two small, short-term contracts (i.e., a few months of fixed-term mandate contracts).

These experiences were positively lived and reinvigorated their optimism. Unlike the precedent subtype (i.e., laborious improvement) they felt that they still had a choice between returning to their old career and moving on to a new one, which signals that their career change was still in elaboration.

Nancy (41), a British librarian forced to change career due to labor market saturation, is an emblematic case of meaningful recovery. When we met her at T1, she was unemployed and was looking for job opportunities mainly as an educational manager. As a mother and nonnative, she encountered difficulties in finding employment or even an internship: “I’m open to doing internships and things like that. But they’re not available…. Even in [Anglo-Saxon countries], there are things called ‘retrainships’ for women … to get back to work … but I don’t find these opportunities in Switzerland” (T1). A year later, her OC situation had not changed. However, she had just finished a temporary, fixed-term contract as an archivist. Although it was short and temporary, she perceived this experience as important and meaningful:

“It was something new for me, so I learned something … to have colleagues.… For … self-esteem and all that, it was positive to work. Even if … it wasn’t so much in my field … it was cool….It was such a great experience... I'm so happy to have had the opportunity to work at [archives]” (T2).

This brief experience reinvigorated her sense of control: “I’ve had difficult experiences, but at the same time, I’ve succeeded in a way and overcome obstacles. I’ve already done it once … so I can do it again” (T2). Nancy was still elaborating her career change at T2. Indeed, she was considering alternative career options but at the same time did not give up on the idea of remaining a librarian: “I’m in a bit of the same situation.… I’ve come back to the beginning now because I’m still looking for work … [in] the library world and archives [and] in program management, like I was last year” (T2). She felt a bit more optimistic: “I don’t know… if I’ll succeed… [in finding a new job]. Maybe with a year’s experience, [I feel] a bit more positive.” (T2).

#### Meaningful Explorations

For two participants, William and Emilie, the OC was rather stagnant and characterized by a status quo. Their OC unfolding was characterized by no strict change of employment status between T1 and T2. However, they both reported several short work experiences (i.e., few weeks fixed-term on mandate contracts in various sectors) during the year. From their SC perspective, these explorations and microtransitions led to an active and dynamic meaning-making process. They perceived their career change process as still in elaboration and marked by increased self-knowledge and self-development. As in the previous subtype, meaning making characterizes these participants. However, participants in the meaningful-exploration subtype let go of their previous occupation to explore new opportunities. This exploration process was still ongoing, and participants did not have clear and definitive options in mind.

William (41) is an emblematic case of the meaningful-exploration subtype. He was a senior manager in aviation forced to change career due to abrupt dismissal during the COVID pandemic. Although his OC situation was rather stagnant between T1 and T2, William experienced several microtransitions in and out of employment, temporary jobs, and training. At T1, he was unemployed and had just exited a short temporary job: “I found a job with an insurance company … in a call center.… Unfortunately, it didn’t suit me at all. It was a much lower position” (T1). When we met with him at T2, he had just started a part-time job in customer service after having had several temporary jobs: “It was also temporary jobs as a receptionist … at hospitals, in companies…. It gave me opportunities to see something else, to see how companies work, which was … beneficial, but unfortunately, it didn’t lead to permanent positions, either” (T2).

From the SC perspective, William went through an active and dynamic meaning-making process associated with his experimentations and microtransitions. At T1, he was still in doubt: “It takes time. You have to question yourself. There’s a lot of doubt … cogitating going on.… I’m going into a total unknown” (T1). When we met with him at T2, he realized that his experiences over the previous year were opportunities to grow and learn:

I’ve really focused this last year on something completely different from this air tourism business.… I took a step back…. I’ve done lots of different things. I’ve actually grown up in all this. I’ve learned a lot…. I joined a lot of recruitment agencies. I did temp missions…. I took courses … to improve myself. (T2)

Although meaningful and leading to an increased self-knowledge and -confidence, his career change process was still ongoing and in elaboration:

I can only learn…. I can only move forward…. I feel I’m taking the right paths [now] … whether it’s right or wrong, I have a certain confidence, and then I know what I want and what I don’t want, in fact, so [I’m no longer] lost. (T2)

#### Vocational Tenacity

Four participants, Idriss, Selim, Josefa, and Anna, unsuccessfully attempted to return to their former occupation through one or two short internship or training experiences related to the previous career, that they interrupted. Consequently, their OC did not evolve or did so only tentatively, keeping them in a sort of suboptimal status quo. Their SC was characterized by tenacious efforts not to lose their former vocational identity. At the same time, given the unlikelihood of a quick return to their initial occupation, they made career compromises (e.g., accepting temporary or on-call jobs) to save the financial resources necessary to possibly recover their former career. They perceived their career change process as extremely long and increasingly demanding, which reveals dissatisfaction and a deterioration of their SC.

The case for Idriss (28), a Syrian airplane pilot who fled political insecurity and whose qualification is not recognized in Switzerland, is emblematic of vocational tenacity. His OC situation did not change between T1 and T2. At T1, he was working occasionally on-call as a waiter in a restaurant while waiting to start a college program to switch his career to management. When we met with him at T2, he had abandoned this program and returned to his side job. Idriss’s OC was also marked by attempts to return to his former occupation. He took several administrative steps to have his pilot diplomas recognized and to fly again. When we met with him a year later, his efforts were not rewarded. Still, he had applied for a grant to earn this equivalence, and in the case of rejection, he considered doing it in a nearby country. Subjectively, he was holding on to his vocational identity as a pilot and perceived the career change as a loss. This was already the case at T1:

The first internship [was] collect the clothes, like the garbage.… I left. I said [to the social worker], “If this will be my job here in Switzerland, after studying for 14 years, it will be better for me to go back to Syria and die there.” I can’t die here every day. (T1)

When we met with him the second time, the feeling of loss was even stronger, pervading every aspect of his life: “I cut my relationship with my [pilot] friends…. Now I’ve lost everything…. I’m losing my family; I’m losing my job; I’m losing my dream job…. If I lose everything, then I’ve left my country for nothing” (T2). Simultaneously, Idriss preferred to make a career compromise and to accept provisional jobs to finance his pilot license: “I’m comfortable finding a job and any job, but a job that I can work temporarily, it’s not a full-time job” (T2). Finally, Idriss perceived his career change as increasingly difficult and never-ending as time passed: “[This project], that’s another six years.… It never stops.” (T2), which adds to the deterioration of his SC. Consequently, he felt career dissatisfaction: “So I feel very disappointed… like a failure.” (T2).

## Discussion

Involuntary career change represents a significant yet understudied transition in the diverse landscape of contemporary career transitions. Importantly, these changes are characterized by their disruptiveness and the unique challenges they pose for individuals (Akkermans et al., 2018; Bidart, 2019; Carless & Arnup, 2011). The aim of this study was to investigate how the process of involuntary career change unfolds over time from subjective and objective perspectives. Through a QLR, we sought to understand not only involuntary career changes’ immediate experiences and impacts but also their evolution over 1 year, addressing their processual complexities (Neale, 2021; Olry-Louis et al., 2022).

Our findings revealed two types of unfolding of involuntary career change: synchronous progressions and asynchronous developments, covering five subtypes of processes. These trajectories illustrate the intricate interplay between OC and SC change processes. Based on our results, two main observations can be made that contribute to a better understanding of involuntary career change experiences: (a) the spectrum of involuntary career change processes and (b) synchronicity as a key temporal element.

### Acknowledging the Spectrum of Involuntary Career Change Processes

Overall, our study confirms that involuntary career change constitutes a multifaceted and challenging career transition. The diversity in our findings underscores the spectrum of involuntary career change processes and calls for an idiosyncratic approach to this phenomenon. At one end of the spectrum, we found individuals experiencing a harmonious deployment, indicating that they successfully regained control and meaning in their careers despite being forced to change. Objectively, this process went hand in hand with a rather linear status progression, including accessing support structures, enrolling in internships or retraining programs, and reintegrating into the labor market. Although in general involuntary career changes imply a risk of lower income and vocational downgrading (Bachmann et al., 2020), participants at this end of the spectrum showed a progression toward better working conditions and possible upward mobility. Even if in a less pronounced way, people in laborious improvement can also be considered as belonging to this end of the spectrum.

In the middle of the spectrum, meaningful recoveries and explorations cover more nuanced situations and processes. A striking finding relates to the contrast between objective and subjective dimensions of involuntary career changes. For these career changers, modest objective steps (e.g., a short internship or work experience) were associated with positive and meaningful subjective experiences. Following these modest objective steps, participants redefined their career and experimented with new career plans. These findings are consistent with the career change literature, which reveals the existence of periods of redefinition and experimentation (Hennekam & Bennett, 2016; Ibarra & Barbulescu, 2010).

At the other end of the spectrum, participants experiencing vocational tenacity went through a loss of control over their careers and struggled to make sense of their experiences. Their OC was characterized by a persistent career insecurity that resulted in no change of status at T2 (e.g., short entry-exit from employment or training, entry into public welfare, obstacles to retraining, aborted internships, or with no stable opportunities, inability to reenter the labor market). This stagnation of their OC in a suboptimal state did not enable them to engage in meaning-making processes, affecting their well-being. In particular, these participants seem stuck in their past vocational identities and try to resist change. The fact that they strongly identify with their past occupation might explain their difficulty in letting go and moving on to a new career, which would be consistent with identity processes addressed in previous research on career change (Hennekam & Bennett, 2016; Kulkarni, 2020).

Finally, irrespective of each type of process’s specific characteristics, all participants appeared to have deliberated on their vocational identity (Savickas, 2020). This deliberation seems to be reflected in three manifestations of identity work (Ibarra & Barbulescu, 2010; Kulkarni, 2020): The identity work of people in synchronous progressions might aim to recreate continuity; that of people in asynchronous developments seems to consist of exploring potential new occupational identities or strengthening the initial vocational identity “against all odds.” These reflections going beyond our research aim, additional research is however needed to further investigate these manifestations of identity work.

### Synchronicities as a Key Temporal Element

Our results confirm that time appears to be a relevant construct for understanding career transitions (Olry-Louis et al., 2022) and involuntary career changes in particular. The analysis of participants’ experiences indicated that these changes are not a sequential and uniform process. Unlike voluntary career change (e.g., Barclay et al., 2011), involuntary career change cannot therefore be conceptualized as a predictable stepwise process. Instead, we advocate considering synchronicity as a key temporal element when studying involuntary career change from a temporal viewpoint.

Synchronization in involuntary career change occurs when individuals experience objective and subjective progression through the change process. Objective improvements generate a subjective sense of progression, which may in turn nurture further OC steps. Synchronized narratives of career change also revealed that despite experiencing some setbacks, individuals remained optimistic and encountered other opportunities that helped them pursue their efforts toward a satisfactory career situation. Simultaneously, individuals envisioned their future with optimistic career plans, anticipating future OC and SC synchronizations. Therefore, synchronization seems to be a key temporal element that allows individuals to bridge past, present, and future in their careers despite being forced to change.

The asynchronized unfolding of career change covers a more complex articulation of objective and subjective careers. Instances of asynchronization reveal a gap between an objective development that is not particularly salient and intensive subjective processes. This situation shows how important the interpretation of events can be in nurturing or hindering a sense of control over one’s career. Asynchronization also might indicate that elaborating an involuntary career change takes time. In situations of meaningful explorations or recovery, time is needed to reflect on and attach meaning to modest working experiences to identify a new career plan. These reflections either help explore new career options (i.e., meaningful explorations) or suggest the possibility of returning to the previous career (i.e., meaningful recovery). In the case of vocational tenacity, the passage of time without any change in the objective situation gradually compromises the possibility of returning to the previous career. For people in vocational tenacity, there seems to be a tension between the feeling that time is taking them away from their former career, and the hope that time will bring them closer to it. In sum, synchronicity reveals various subjective elaborations of time: Time can be either a marker of continuity (synchronous progressions), change, or rupture (asynchronous developments) (Neale, 2021; Olry-Louis et al., 2022).

### Limitations and Perspectives

Although this study makes significant contributions to our understanding of involuntary career change processes, we acknowledge four limitations that pave the way for future research. First, participant recruitment occurred through public and semipublic institutions offering career changers diverse types of support. Frequency of interactions with career professionals varied based on institutional policies, welfare provisions, information accessibility, and regional disparities. These disparities may have influenced participants’ strategies, readiness, and experiences in career change. Our recruitment strategy also excluded possible career changers who were not supported by institutions and who might, therefore, face more challenges in the change process. Researchers could implement alternative recruitment methods to access a more homogeneous population, encompass a wider range of career changers.

Second, a temporal thematic analysis provides a comprehensive processual overview but prevents us delving deeply into the complex subjective experience of each individual undergoing a career change. Indeed, our participants encountered various kinds of life challenges, such as family constraints, diploma recognition procedures, and health deterioration, that uniquely shaped their career change processes. Therefore, research involving a smaller sample and prioritizing an in-depth approach to each individual’s unique experience, such as interpretative phenomenological analysis (e.g., Ahn et al., 2017), could complement our study. Such an approach would also allow us to extend the aims of the present research and explore the range of adaptive processes deployed by workers in face of an involuntary career change.

Third, although we met them within the first year of their career change, at T1 participants were at different points in their change process. These differences could have influenced the way they referred to their career change. For example, individuals with clear career plans at T1 may have offered a more optimistic view than those navigating uncertain career paths. In addition, at T2, some career changes were still ongoing, while others were achieved. Although longitudinal qualitative research involves a perspective shift from a static snapshot to a dynamic view of subjective and objective experiences, it remains framed in a temporal window. Therefore, as Thomson and Holland (2003) noted, achieving analytical closure in qualitative longitudinal research is highly challenging. The temporal window of our study, although it provided valuable insights, was rather modest and warrants cautious interpretations of our findings because previous or further interview waves could tinge our results. Extending longitudinal studies, upstream and downstream of the career change process, would then provide a comprehensive understanding of this process (Neale, 2021).

Fourth, our typologization procedure did not take into account participants’ sociodemographic and biographical characteristics, such as gender, age, reasons for change, and type of support. These characteristics might impact the change process and some profiles of participants might be overrepresented in a given type of career change process. Further investigations could provide insights into possible associations between participants’ characteristics and involuntary career change unfolding.

### Practical Implications

Our study has several implications for career intervention. A key insight is the need to move beyond a stereotypical view of involuntary career change sequences, that is, exiting work, retraining, and returning to work. Instead, we argue that recognizing and validating a wider array of possible experiences of involuntary career change is critical to implementing career interventions successfully. Indeed, depending on the identified types of unfolding, distinct forms of support could be implemented. For synchronous progressions, career professionals might help individuals consolidate their career project. This consolidation could secure decent work and sustainable careers and prevent further career shocks (Akkermans et al., 2018). When individuals experience asynchronous developments, support should consider their subjective experiences. Career support could nurture meaning making, explorations, and microtransitions but also promote more tangible objective progress. When individuals engage in volunteering, self-development courses, short online training, temporary contracts, internships, or associative advocacy, career counselors might help them valorize skills they developed in these activities and translate them into secure working positions. When subjective career developments imply dissatisfaction and vocational tenacity, individuals may need more time to see their tenacity fructify or identify meaningful alternative career options. Meanwhile, intensive psychological support might help them face the potential career loss and, if needed, “let go” of their lost vocational identity and move on to a new career plan that is as meaningful and secure as possible (Kulkarni, 2020).

## Conclusion

Our study emphasized the idiosyncratic nature of involuntary career changes. In particular, we highlighted the existence of a variety of unfolding of involuntary career change, ranging from positive progressions to more modest developments and deteriorations of the situation. Our findings also showed the importance of considering the degree of synchronicity between the OC and SC of career changers; a discrepancy between the objective evolution of the situation and its subjective experience could indicate more challenging involuntary career change processes. Finally, the present study is one of the few (if not the only) in the field of career development to implement QLR, which appears to be a promising methodological strategy for addressing the characteristics of contemporary careers and career transitions.

## Appendix

### Appendix A: Time 1 & Time 2 Interview Guidelines

#### Time 1

**1. Sociodemographic information:** age, living situation, family situation, education level, country of origin
**2. Career path:** Can you tell me what occupations you have held throughout your career and how long they lasted ? In the past, what made you change jobs? What life events have affected your career path? Here, you can see images that represent career paths. Which image would best describe the evolution of your career path? Which image best describes your sense of control over your journey? How satisfied were you in your last job? What is your current professional situation?
**3. Involuntary career change process:** Can you tell me what brought you to this point professionally? What are the reasons for your career change? How has the pandemic situation influenced the transition process? To what extent do you feel that you have been able to choose this career change? To what extent did you expect this career change? What is the likelihood that you will be able to return to your previous occupation? What did you say to yourself when you learned that you had to change careers? What did you feel at that moment? What links do you perceive between the job you had before and your current career plan?
**4. Closing reflections**: Is there any additional information that is important for understanding your career change? Do you have any questions for me? How do you feel after this interview?

#### Time 2

**1. Sociodemographic information**: Changes in age, living situation, family situation, and legal status
**2. Career path:** It has been a year now since we parted, so could you tell me where you stand since we spoke? How has your career change situation evolved since our first discussion? To what extent did you expect things to turn out as they did? One year after our first meeting, how would you assess your situation and your career change? How did the lifeline exercise go for you? Can you explain your lifelines to me? You’ve drawn two different lines. What links do you see between them? Imagine that this line continues into the future. What shape will it take? Try to project yourself into the distant future (e.g., 10 years from now). Who do you think you’ll be at that time (professionally)? 10 years from now, what would your ideal situation be, and what situation would you like to avoid? What do you retain from this lifeline exercise?
**3. Involuntary career change process:** Looking back, to what extent do you feel you were able to choose this career change? Today, if it were possible, how tempted would you be to return to your former occupation? When we last met, you told me about your feelings about the changeover (mention their case specifically). How have these feelings changed over the past year?
**4. Closing reflections**: Is there any additional information that is important for understanding your career change? Do you have any questions for me? How do you feel after this interview? What was it like to take part in this research project?

## References

[bibr1-10690727241289126] Ahn J. Dik B. J. Hornback R. (2017). The experience of career change driven by a sense of calling: An Interpretative Phenomenological Analysis approach. Journal of Vocational Behavior, 102, 48–62. 10.1016/j.jvb.2017.07.003

[bibr2-10690727241289126] Akkermans J. Seibert S. E. Mol S. T. (2018). Tales of the unexpected: Integrating career shocks in the contemporary careers literature. SA Journal of Industrial Psychology, 44(a1503), 1–10. 10.4102/sajip.v44i0.1503

[bibr3-10690727241289126] Arvinen-Barrow M. Degrave K. Pack S. Hemmings B. (2018). Transitioning out of professional sport: The psychosocial impact of career-ending non-musculoskeletal injuries among male cricketers from England and Wales. Journal of Clinical Sport Psychology, 13(4), 629–644. 10.1123/jcsp.2017-0040

[bibr4-10690727241289126] Audulv Å. Hall E. O. C. Kneck Å. Westergren T. Fegran L. Pedersen M. K. Aagaard H. Dam K. L. Ludvigsen M. S. (2022). Qualitative longitudinal research in health research: A method study. BMC Medical Research Methodology, 22(1), 255. 10.1186/s12874-022-01732-4 36182899 PMC9526289

[bibr5-10690727241289126] Bachmann R. Bechara P. Vonnahme C. (2020). Occupational mobility in Europe: Extent, determinants and consequences. De Economist, 168(1), 79–108. 10.1007/s10645-019-09355-9

[bibr6-10690727241289126] Baldridge D. C. Kulkarni M. (2017). The shaping of sustainable careers post hearing loss: Toward greater understanding of adult onset disability, disability identity, and career transitions. Human Relations, 70(10), 1217–1236. 10.1177/0018726716687388

[bibr7-10690727241289126] Barclay S. R. Stoltz K. B. Chung Y. B. (2011). Voluntary midlife career change: Integrating the transtheoretical model and the life-span, life-space approach. The Career Development Quarterly, 59(5), 386–399. 10.1002/j.2161-0045.2011.tb00966.x

[bibr8-10690727241289126] Bidart C. (2019). How plans change: Anticipation, interferences, and unpredictabilities. Advances in Life Course Research*, *41, Article 100254. 10.1016/j.alcr.2018.10.007 36738030

[bibr50-9106907272418] Brazier C. É. Masdonati J. Borges A. Fedrigo L. Cerantola M. (2024). Drivers of involuntary career changes: A qualitative study of push, pull, anti-push, and anti-pull factors. Journal of Career Development, 51(3), 303–326. 10.1177/08948453241246720

[bibr9-10690727241289126] Carless S. A. Arnup J. L. (2011). A longitudinal study of the determinants and outcomes of career change. Journal of Vocational Behavior, 78(1), 80–91. 10.1016/j.jvb.2010.09.002

[bibr10-10690727241289126] De Vos A. Jacobs S. Verbruggen M. (2021). Career transitions and employability. Journal of Vocational Behavior, 126, Article, 103475. 10.1016/j.jvb.2020.103475

[bibr11-10690727241289126] Farr J. Nizza I. E. (2019). Longitudinal interpretative phenomenological analysis (LIPA): A review of studies and methodological considerations. Qualitative Research in Psychology, 16(2), 199–217. 10.1080/14780887.2018.1540677

[bibr12-10690727241289126] Federal Statistical Office . (2020). Enquête suisse sur la population active (ESPA): La mobilité professionnelle en Suisse. FSO. https://www.bfs.admin.ch/bfs/en/home/statistics/work-income/employment-working-hours/economically-active-population/occupational-mobility.assetdetail.14667036.html

[bibr13-10690727241289126] Fouad N. A. Bynner J. (2008). Work transitions. American Psychologist, 63(4), 241–251. 10.1037/0003-066X.63.4.241 18473609

[bibr49-9106907272420] Frésard C. É. Parmentier M. Oliveira Borges A. Masdonati J. (2022). Les reconversions professionnelles involontaires : Bilan de la première vague d’entretiens. [Involuntary career changes: Technical report from the first wave of interviews]. Université de Lausanne. 10.13140/RG.2.2.23874.25285

[bibr14-10690727241289126] Froidevaux A. Hirschi A. Wang M. (2018). Identity incongruence and negotiation in the transition from work to retirement: A theoretical model. Organizational Psychology Review, 8(4), 228–255. 10.1177/2041386619830754

[bibr15-10690727241289126] Gardiner J. Stuart M. MacKenzie R. Forde C. Greenwood I. Perrett R. (2009). Redundancy as a critical life event: Moving on from the Welsh steel industry through career change. Work, Employment and Society, 23(4), 727–745. 10.1177/0950017009344917

[bibr16-10690727241289126] Haynie J. M. Shepherd D. (2011). Toward a theory of discontinuous career transition: Investigating career transitions necessitated by traumatic life events. Journal of Applied Psychology, 96(3), 501–524. 10.1037/a0021450 21090884

[bibr17-10690727241289126] Hennekam S. Bennett D. (2016). Involuntary career transition and identity within the artist population. Personnel Review, 45(6), 1114–1131. 10.1108/Pr-01-2015-0020

[bibr18-10690727241289126] Hermanowicz J. C. (2013). The longitudinal qualitative interview. Qualitative Sociology, 36(2), 189–208. 10.1007/s11133-013-9247-7

[bibr19-10690727241289126] Ibarra H. Barbulescu R. (2010). Identity as narrative: Prevalence, effectiveness, and consequences of narrative identity work in macro work role transitions. The Academy of Management Review, 35(1), 135–154. 10.5465/Amr.2010.45577925

[bibr20-10690727241289126] Kulkarni M. (2020). Holding on to let go: Identity work in discontinuous and involuntary career transitions. Human Relations, 73(10), 1415–1438. 10.1177/0018726719871087

[bibr21-10690727241289126] Lent R. W. Wang R. J. Cygrymus E. R. Moturu B. P. (2023). Navigating the multiple challenges of job loss: A career self-management perspective. Journal of Vocational Behavior*, *146, Article 103927. 10.1016/j.jvb.2023.103927

[bibr23-10690727241289126] Lipshits-Braziler Y. Gati I. (2019). Facilitating career transitions with coping and decision-making approaches. In Maree J. G. (Ed.), Handbook of innovative career counselling (pp. 139–156). Springer. 10.1007/978-3-030-22799-9_9

[bibr48-9106907272415] Masdonati J. Fournier G. Lahrizi I. Z. (2017). The reasons behind a career change through vocational education and training. International Journal for Research in Vocational Education and Training, 4(3), 249–269. 10.13152/IJRVET.4.3.4

[bibr47-9106907272416] Masdonati J. Frésard C. É. Parmentier M. (2022). Involuntary career changes: A lonesome social experience. Frontiers in Psychology, 13(899051). 10.3389/fpsyg.2022.899051 PMC9202451 35719552

[bibr24-10690727241289126] McCoy L. K. (2017). Longitudinal qualitative research and interpretative phenomenological analysis: Philosophical connections and practical considerations. Qualitative Research in Psychology, 14(4), 442–458. 10.1080/14780887.2017.1340530

[bibr25-10690727241289126] Medici G. Tschopp C. Grote G. Hirschi A. (2020). Grass roots of occupational change: Understanding mobility in vocational careers. Journal of Vocational Behavior*, *122, Article 103480. 10.1016/j.jvb.2020.103480

[bibr26-10690727241289126] Motulsky S. L. (2010). Relational processes in career transition: Extending theory, research, and practice. The Counseling Psychologist, 38(8), 1078–1114. 10.1177/0011000010376415

[bibr27-10690727241289126] Mulhall S. (2014). (Re)constructing career strategies after experiencing involuntary job loss. Journal of Change Management, 14(4), 453–474. 10.1080/14697017.2014.978534

[bibr28-10690727241289126] Murtagh N. Lopes P. N. Lyons E. (2011). Decision making in voluntary career change: An other-than-rational perspective. The Career Development Quarterly, 59(3), 249–263. 10.1002/j.2161-0045.2011.tb00067.x

[bibr29-10690727241289126] Neale B. (2021). The craft of qualitative longitudinal research. Sage.

[bibr30-10690727241289126] Neale B. Tarrant A. (2024). The dynamics of young fatherhood: Understanding the parenting journeys and support needs of young fathers. Policy Press.

[bibr31-10690727241289126] Olry-Louis I. Cocandeau-Bellanger L. Fournier G. Masdonati J. (2022). Temporality: A fruitful concept for understanding, studying, and supporting people in transition. The Career Development Quarterly, 70(4), 256–270. 10.1002/cdq.12306

[bibr32-10690727241289126] Palic D. Nardon L. Hari A. (2023). Transnational sensemaking narratives of highly skilled Canadian immigrants’ career change. Career Development International, 28(4), 392–405. 10.1108/CDI-06-2022-0182

[bibr33-10690727241289126] Ponterotto J. G. (2005). Qualitative research in counseling psychology: A primer on research paradigms and philosophy of science. Journal of Counseling Psychology, 52(2), 126–136. 10.1037/0022-0167.52.2.126

[bibr34-10690727241289126] Saldaña J. (2003). Longitudinal qualitative research: Analyzing change through time. AltaMira Press.

[bibr35-10690727241289126] Savickas M. L. (2000). Renovating the psychology of careers for the twenty-first century. In Collin A. Young R. A. (Eds.), The future of career (pp. 53–68). Cambridge University Press.

[bibr36-10690727241289126] Savickas M. L. (2002). Career construction, a developmental theory of vocational behavior. In Brown D. (Ed.), Career choice and development (4th ed., pp. 149–205). Wiley.

[bibr37-10690727241289126] Savickas M. L. (2020). Career construction theory and counseling model. In Lent R. W. Brown S. D. (Eds.), Career development and counseling: Putting theory and research to work (3rd ed., pp. 155–200). Wiley.

[bibr38-10690727241289126] Schoon I. Heckhausen J. (2019). Conceptualizing individual agency in the transition from school to work: A social-ecological developmental perspective. Adolescent Research Review, 4(2), 135–148. 10.1007/s40894-019-00111-3

[bibr39-10690727241289126] Sheard L. Marsh C. (2019). How to analyse longitudinal data from multiple sources in qualitative health research: The pen portrait analytic technique. BMC Medical Research Methodology, 19(1), 169. 10.1186/s12874-019-0810-0 31375082 PMC6679485

[bibr40-10690727241289126] Sim J. Saunders B. Waterfield J. Kingstone T. (2018). Can sample size in qualitative research be determined a priori? International Journal of Social Research Methodology, 21(5), 619–634. 10.1080/13645579.2018.1454643

[bibr41-10690727241289126] Sullivan S. E. Al Ariss A. (2021). Making sense of different perspectives on career transitions: A review and agenda for future research. Human Resource Management Review, 31(1). 10.1016/j.hrmr.2019.100727

[bibr42-10690727241289126] Thomson R. Holland J. (2003). Hindsight, foresight and insight: The challenges of longitudinal qualitative research. International Journal of Social Research Methodology, 6(3), 233–244. 10.1080/1364557032000091833

[bibr43-10690727241289126] Treanor M. C. Patrick R. Wenham A. (2021). Qualitative longitudinal research: From monochrome to technicolour. Social Policy and Society, 20(4), 635–651. 10.1017/S1474746421000270

[bibr44-10690727241289126] Vogl S. Zartler U. Schmidt E. M. Rieder I. (2018). Developing an analytical framework for multiple perspective, qualitative longitudinal interviews (MPQLI). International Journal of Social Research Methodology, 21(2), 177–190. 10.1080/13645579.2017.1345149

[bibr45-10690727241289126] Wise A. J. Millward L. J. (2005). The experiences of voluntary career change in 30-somethings and implications for guidance. Career Development International, 10(5), 400–417. 10.1108/13620430510615328

[bibr46-10690727241289126] Zacher H. (2019). Berufliche Veränderungen: Wenn Erwerbstätige sich neu orientieren. [Career changes: When employed persons reorient themselves]. In Kauffeld S. Spurk D. (Eds.), Handbuch Karriere und Laufbahnmanagement (pp. 1–23). Springer. 10.1007/978-3-662-45855-6_14-1

